# Altered Functional Connectivity Dynamics Serving Cognitive Flexibility in Regular Cannabis Users

**DOI:** 10.1111/adb.70023

**Published:** 2025-03-05

**Authors:** Kellen M. McDonald, Mikki Schantell, Jason A. John, Anna T. Coutant, Ryan Glesinger, Lucy K. Horne, Hannah J. Okelberry, Seth D. Springer, Christine M. Embury, Yasra Arif, Tony W. Wilson

**Affiliations:** ^1^ Institute for Human Neuroscience Boys Town National Research Hospital Omaha Nebraska USA; ^2^ Department of Pharmacology & Neuroscience Creighton University Omaha Nebraska USA; ^3^ College of Medicine University of Nebraska Medical Center (UNMC) Omaha Nebraska USA

**Keywords:** attention network, magnetoencephalography, MEG, oscillations, task switch

## Abstract

Despite its widespread use and popularity, cannabis is known to impact higher order cognitive processes such as attention and executive function. However, far less is known about the impact of chronic cannabis use on cognitive flexibility, a component of executive function, and this is especially true for the underlying functional connectivity dynamics. To address this, we enrolled 25 chronic cannabis users and 30 demographically matched non‐users who completed an interview probing current and past substance use, a urinalysis to confirm self‐reported substance use and a task‐switch cognitive paradigm during magnetoencephalography (MEG). Time‐frequency windows of interest were identified using a data‐driven statistical approach, and spectrally specific neural oscillatory responses were imaged using a beamformer. The resulting maps were grand‐averaged across all participants and conditions, and the peak voxels in these maps of neural oscillatory activity were used as seeds to compute connectivity using a whole‐brain cortical‐coherence approach. Whole‐brain neural switch cost connectivity maps were then computed by subtracting the connectivity map for the no‐switch condition from that of the switch condition per participant. These switch cost functional connectivity maps were then correlated with the behavioural switch cost per group and probed for group differences in the neuro‐behavioural associations. Our behavioural results indicated that all participants had slower reaction times during switch compared to no‐switch trials. Regarding the MEG data, cannabis users exhibited altered associations between functional connectivity switch costs and behavioural switch costs along pathways connecting visual cortices and regions in the ventral attention network, within the theta, alpha and gamma frequency ranges. These results indicate modified multispectral associations between functional connectivity and behavioural switch costs among visual cortices and key brain regions underlying executive function in cannabis users.

## Graphical abstract

To examine the effects of chronic cannabis use on the oscillatory dynamics serving cognitive flexibility, users and non‐users completed a task‐switching paradigm during magnetoencephalography. Users displayed altered associations between functional connectivity switch costs and behavioural switch costs along pathways linking visual cortices and ventral attention network regions, particularly in the gamma range.

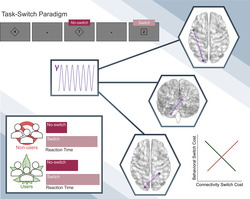

## 1 Introduction

Cannabis is one of the most widely used psychoactive substances among adults in the United States. The number of midlife cannabis users significantly increased from 2021 to 2022, going from 24.9% to 27.9% for use within the year and 15.8% to 17.3% for use within the month [1, 2]. These upward trends are likely to continue as cannabis becomes more accessible and publicly accepted. Despite broad use and social acceptance, studies have shown detriments to higher‐order cognitive processes among both acute and long‐term cannabis users [3, 4, 5, 6], which can negatively impact various aspects of daily life [7]. In fact, meta‐analyses of cannabis‐related neurocognitive effects have generally concluded that cognitive deficits are detectable and persist long after acute use [8, 9, 10, 11], with impairments in attention, memory, learning, and executive function showing especially strong residual deficits in chronic cannabis users.

Cognitive flexibility, which is often probed using set‐shifting or task‐switch paradigms, is essential to executive function. Broadly, cognitive flexibility enables one to selectively switch mental processes due to environmental or situational changes to produce task‐appropriate behavioural responses [12]. Because a multitude of cognitive operations such as sustained attention and inhibition contribute to cognitive flexibility, a wide range of brain regions and networks have been associated with cognitive flexibility due to the different cognitive operations and task subcomponents involved [12]. The fronto‐parietal network, which has been implicated in functional MRI (fMRI) and magnetoencephalography (MEG) task switching studies, is generally thought of as a flexible hub for the effective control of cognition [13]. It integrates with other systems to support processes such as cognitive flexibility, including the dorsal and ventral attention networks (DAN and VAN, respectively) and salience network [13, 14, 15]. In MEG studies, significant changes in the alpha, beta, gamma, and theta frequency ranges have been reported within nodes of these attention‐related networks during switch relative to no‐switch trials [14, 16].

Our previous work focusing on cannabis and cognitive flexibility revealed weaker theta oscillations during switch relative to no‐switch trials in regions of the prefrontal cortex in chronic users compared to non‐users, with weaker activity scaling with greater cannabis use involvement among users [16]. However, these analyses were limited to group differences in the strength of specific regional alterations, as opposed to the connectivity among brain regions exhibiting robust oscillatory activity across all participants. Still, this was in line with previous MEG studies of chronic cannabis use, which have found altered theta activity during attention‐related tasks in the absence of task performance differences [17, 18]. Similarly, in animal models of cannabis use, altered theta and gamma oscillations have been widely reported and are thought to reflect the modulation of cannabinoid (CB)‐1 receptors [19, 20, 21]. CB_1_ receptors are distributed throughout the cortex and heavily concentrated in frontal, limbic, and cerebellar regions [22, 23]. Δ9‐tetrahydrocannabinol, the main psychoactive component in cannabis, is an agonist of endocannabinoid CB_1_ receptors [24], which due to their widespread availability have been linked to changes in theta and gamma oscillatory activity and executive functioning [25, 26, 27]. Unsurprisingly, alterations to these frequency bands are of major interest in the cannabis MEG literature [28, 29, 30, 31].

In the fMRI literature, altered connectivity patterns between cannabis users and non‐users during both resting‐state and cognitively demanding tasks have been reported [32, 33, 34], with Harding et al. [35] interpreting greater connectivity between frontal and occipitoparietal cortices as an indicator of compensatory processing among long‐term users during attentional processing. While highly informative, these previous fMRI studies are not able to provide insight on the precise temporal or spectral aspects of cannabis‐related connectivity differences. As such, in the current study, we examined multispectral connectivity changes during switch relative to no‐switch trials (i.e., switch costs) in chronic cannabis users and non‐user controls. We were particularly interested in the relationship between connectivity and behavioural signatures of switch costs and how these relationships may be altered in chronic cannabis users. Thus, participants in each group completed a validated task‐switch paradigm during MEG, and we computed multispectral functional maps per condition, which were then used to compute switch cost maps (i.e., switch – no‐switch trials), and these were examined with respect to behavioural switch costs. Given the previous findings noted above, we hypothesized that chronic cannabis users would exhibit altered theta range functional connectivity (FC) compared to non‐users during task switching, along with group differences in the gamma, alpha, and beta ranges related to the neural switch cost.

## 2 Methods

### 2.1 Participants

We enrolled 55 participants between the ages of 19 and 60 years. Full demographics are provided in the results section. Cannabis users were required to have used cannabis at least three times per week for three years or more, and non‐users were recruited from a larger study in which they indicated that they did not use cannabis or other illicit substances. Participants who reported a history of experimental use for a short time during youth were not excluded from the control group. Exclusion criteria for the study included any diagnosed neurological or psychiatric disorder, any medical illness associated with CNS dysfunction, history of head trauma, current substance use disorder other than cannabis use disorder in the user group, the presence of metallic implants that could affect the MEG and MRI data acquisition or be a safety concern, and current pregnancy. The local Institutional Review Board reviewed and approved this investigation. Participants provided written informed consent following detailed description of the study, and the study was conducted in accordance with the Declaration of Helsinki. Participants completed the study over the course of 2–3 visits. During the first visit, participants underwent informed consent, a medical history intake, and a neuropsychological assessment. During the second visit, participants completed the MEG recording, substance use interview, and self‐report assessments. Participants had the option of completing the MRI scan during the second visit or during a separate third visit.

### 2.2 Substance Use Assessments

All participants underwent a thorough structured interview regarding their current (within the past 12 months) and past (prior to the past 12 months) substance use history using Module E of the Structured Clinical Interview for the Diagnostic and Statistical Manual, 5th Edition (SCID‐5), the NIDA Quick Screen (Version 1) and the NIDA‐Modified Alcohol, Smoking, and Substance Involvement Screening Test (NIDA‐ASSIST; Version 2). Participants also completed self‐report questionnaires including the Alcohol Use Disorders Identification Test (AUDIT), and cannabis users completed the Cannabis Use Disorders Identification Test–Revised (CUDIT‐R) and the Daily Sessions, Frequency, Age of Onset, and Quantity of Cannabis Use (DFAQ‐CU) Inventory. Cannabis users and non‐users also provided a sample for urinalysis to confirm that participants in the user group had not recently used substances other than cannabis and that controls had not recently used cannabis or any recreational substances. At the beginning of each visit, all participants were subjected to a breathalyser test and were asked about their past‐24‐h alcohol, tobacco, and recreational substance use and whether it was different from their usual use. Note there were no group differences in past‐24‐h use of alcohol (*p* = 0.30) or tobacco (*p* = 0.51). Because we were primarily interested in the impact of chronic cannabis use on cognitive flexibility, participants who were under the influence of alcohol or any other substances or had used cannabis within 8 h of their visit were rescheduled.

### 2.3 Neuropsychological Assessment

Cognitive function was measured using a neuropsychological battery that assessed premorbid function and functionality across seven domains (i.e., learning, memory, executive function, attention, processing speed, language and motor dexterity). The battery included the following tests for each domain: *learning* (Wechsler Memory Scale [WMS‐III] Logical Memory Initial Recall [36], California Verbal Learning Test [CVLT‐II] Learning Trials 1–5 [37]), *memory* (CVLT‐II Delayed Recall and Recognition Discriminability Index [37], WMS‐III Logical Memory II Delayed Recall [36]), *executive function* (Comalli Stroop Test Interference Trial [38] and Trail Making Test Part B [39]), *processing speed* (Comalli Stroop Test Colour and Word Trials [38], Wechsler Adult Intelligence Scale (WAIS‐III) Digit Symbol Coding [40] and Trail Making Part A [39]), *attention* (WAIS‐III Letter Number Sequencing [40], WAIS‐III Digit Span Forward and Backward Trials [40] and CVLT‐II Trial 1 [37]), *language* (phonemic verbal fluency and semantic verbal fluency [39]) and *motor dexterity* (Grooved Pegboard, Dominant and Non‐Dominant Hands [39, 41]). Further, *premorbid function* was assessed using the Wide Range Achievement Test 4 (WRAT‐4) Word Reading [42]. Demographically corrected scores for each assessment were obtained using published normative data [37, 38, 39, 40, 41, 42] and were computed to z‐scores. Domain composite scores were computed by averaging the z‐scores of assessments that comprised each respective cognitive domain. Participants also completed the Beck Depression Inventory‐II (BDI‐II; [43]) as a measure of depression.

### 2.4 MEG Experimental Paradigm

**FIGURE 1:**
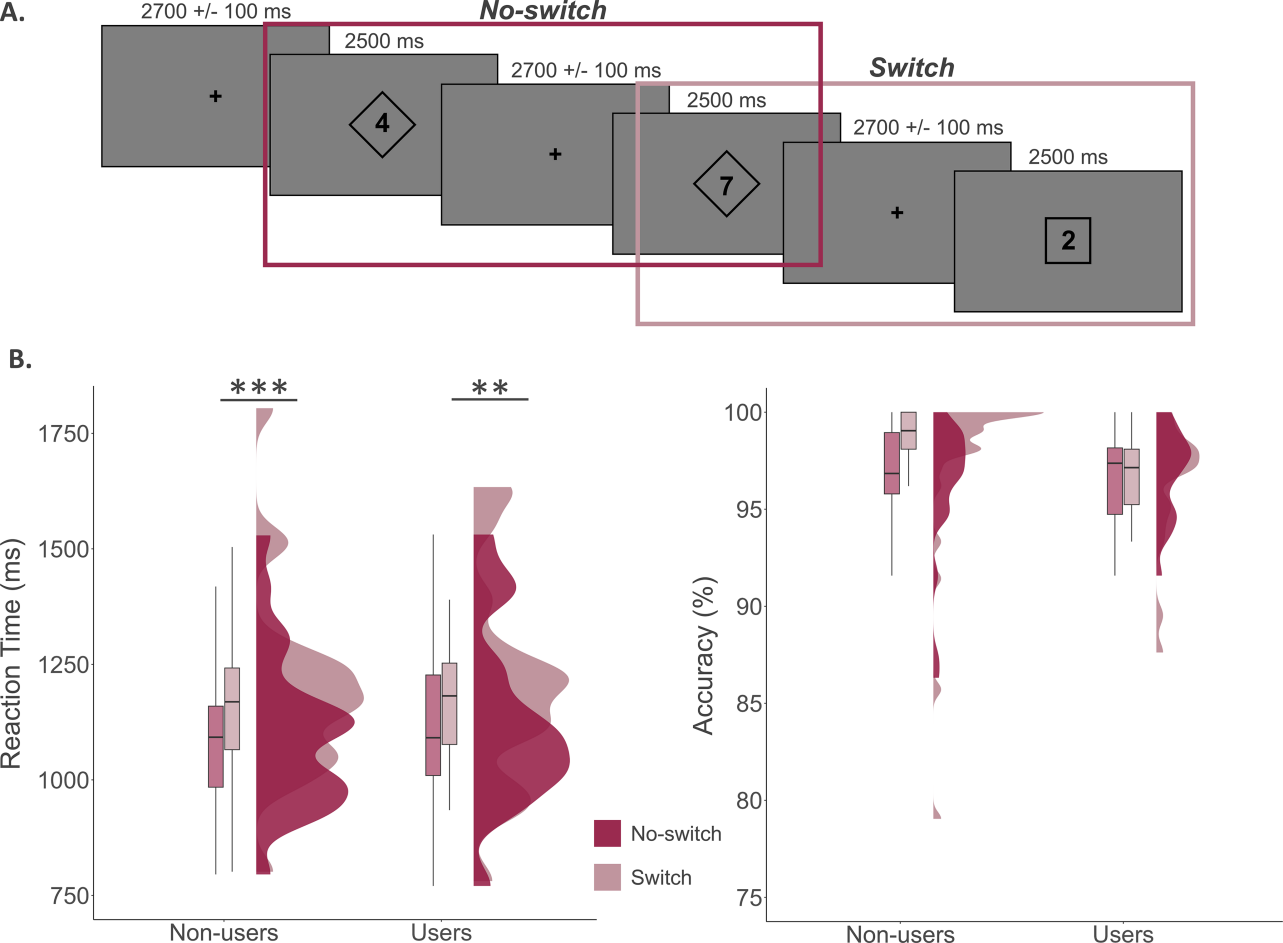
Task‐switch experimental paradigm and behavioural results. (A) Example stimuli with the first digit shown after the initial fixation cross to be within a diamond and the next digit to also be within a diamond, repeating the previous rule (i.e., a no‐switch trial). Conversely, the third digit is within a square, indicating a switch from the previous rule (i.e., switch trial). (B) Behavioural reaction time (left) and accuracy (right) data. There was a significant conditional effect, with all participants responding slower during the switch relative to no‐switch trials (**p < 0.005; ***p < 0.001). No other effects were significant.

Participants underwent a 17.5‐min task switching experiment (Figure 1), which has been validated and described in previous work [14]. Participants were seated in a magnetically shielded room and were instructed to fixate on a centrally presented crosshair with a variable ISI (range: 2600–2800 ms) followed by the presentation of a single digit ranging from 1 to 9 (excluding five) in either a square or a diamond of equal dimensions for 2500 ms (Figure 1A). The instructions were counterbalanced across participants to eliminate any potential confounding effects. For one set of instructions, participants were told that a number surrounded by a square indicated they should respond as to whether the number was less than (index finger) or greater than (middle finger) five. Conversely, if the number was surrounded by a diamond, they should respond as to whether the number was odd (index finger) or even (middle finger). In the other set of instructions, the meaning of the square versus diamond was switched. There were 200 pseudo‐randomized trials, equally balanced between squares and diamonds, with half of the trials repeating the previous trial's rule (i.e., no‐switch) and the other trials switching from the previous trial's rule (i.e., switch). Each trial lasted 5100–5300 ms. Reaction time and accuracy measures were collected and used for behavioural analysis.

### 2.5 MEG and MRI Data Acquisition

Functional MEG data were collected using a MEGIN MEG system (Helsinki, Finland) equipped with 306 sensors (204 planar gradiometers, 102 magnetometers) using a 1 kHz sampling rate and an acquisition bandwidth of 0.1–330 Hz in a one‐layer magnetically shielded room with active shielding engaged. Prior to MEG acquisition, four coils were attached to the participant's head and localized along with fiducial and scalp surface points using a three‐dimensional digitizer (FASTRAK, Polhemus Navigator Sciences, Colchester, Vermont). Once the participants were positioned for MEG recording, an electric current with a unique frequency label (e.g., 322 Hz) was fed to each of the four coils, thus inducing a measurable magnetic field and thereby allowing each coil to be localized in reference to the MEG sensor array throughout the recording session. High‐resolution structural images were also collected using a T1‐weighted three‐dimensional 1‐mm isotropic MPRAGE sequence [TR = 2.3 s, TE = 2.98 ms, flip angle = 9°, FOV = 256 mm] on a Siemens Prisma 3‐T scanner with a 64‐channel head coil.

### 2.6 MEG and MRI Processing

MEG and MRI data processing closely followed previously reported pipelines [14, 44, 45]. The structural MRI data were aligned parallel to the anterior and posterior commissures and transformed into standardized space. MEG data were subjected to environmental noise reduction and corrected for head motion using the signal space separation method with a temporal extension [46]. Only data from the 204 planar gradiometers were used for further analysis. All MEG and MRI data were further processed in BESA (Research: Version 7.0; MRI: Version 2.0; Statistics: Version 2.0). Cardiac and ocular artefacts were removed from the MEG data using signal space projection (SSP; [47]).

### 2.7 MEG Time‐Frequency Transformation and MEG Sensor‐Level Statistics

The continuous magnetic time series was then filtered between 0.5 and 200 Hz, and a 60‐Hz notch filter was applied. Epochs were 3500 ms, with the baseline extending from −500 to 0 ms prior to visual stimulus onset. Only trials with correct responses were considered for further analysis. Epochs containing artefacts were rejected using a fixed threshold method that was set per participant and supplemented with visual inspection. Briefly, in MEG, the raw signal amplitude is strongly affected by the distance between the brain and the MEG sensor array, as the magnetic field strength falls off exponentially as the distance from the current source (i.e., brain) to the sensor array increases. To account for this source of variance across participants, as well as other sources of variance (e.g., head size), we used an individualized threshold based on the signal distribution for both amplitude and gradient to reject artefacts. Cannabis users and non‐users did not differ in their amplitude (*t* = 0.16, *p* = 0.876; cannabis users: *M* = 1651.25 fT/cm, *SD* = 615.61; non‐users: *M* = 1686.96 fT/cm, *SD* = 836.01) and gradient cut‐offs (*t* = 0.24, *p* = 0.809; cannabis users: *M* = 544.00 fT/(cm*ms), *SD* = 332.12; non‐users: *M* = 571.09 fT/(cm*ms), *SD* = 390.10), nor in the number of epochs retained for analysis in the switch (*t* = 0.44, *p* = 0.662; cannabis users: *M* = 87.85, *SD* = 3.73; non‐users: *M* = 88.57, *SD* = 6.36) and no‐switch conditions (*t* = −0.23, *p* = 0.821; cannabis users: *M* = 87.75, *SD* = 3.68; non‐users: *M* = 87.48, *SD* = 4.10).

We then transformed the artefact‐free epochs into the time‐frequency domain (resolution: 2 Hz, 25 ms; 4–100 Hz) using complex demodulation [48, 49]. Each sensor's spectral power estimations were averaged over trials to produce time‐frequency plots of mean spectral density and then normalized by the baseline power of each respective bin, which was calculated as the mean power from −500 to 0 ms. Next, we determined time‐frequency windows for source analysis based on a data‐driven approach that used paired‐sample *t*‐tests against baseline across all participants for each pixel in the spectrograms, with the significant pixels being subjected to cluster‐based permutation testing (10,000 permutations) to control for Type 1 error. The initial cluster threshold was *p* < 0.005.

### 2.8 MEG Source Imaging

The time‐frequency resolved source images were computed using the dynamic imaging of coherent sources (DICS) beamformer to image oscillatory activity in the time‐frequency windows that survived the sensor‐level statistical analysis described above [50, 51, 52]. We used active task and pre‐stimulus baseline periods of equal duration and bandwidth [53] to compute noise‐normalized source power per voxel, with the resulting pseudo‐*t* units reflecting power differences (i.e., active versus baseline) per voxel (resolution: 4 × 4 × 4 mm). The resulting beamforming maps were then transformed into standardized space and spatially resampled by applying the same transform that was applied to the native space structural images per participant. These images were then grand averaged separately for each time‐frequency window of interest across all participants and conditions and separately between cannabis users and non‐users to visually depict differences in power as a function of cannabis use.

### 2.9 Functional Connectivity (FC)

To probe FC, the peak voxels from the grand averaged beamformer images were used as seed voxels for the calculation of a coherence beamformer using the DICS approach [51]. Switch and no‐switch images were computed separately, and whole‐brain neural switch cost maps were computed by subtracting no‐switch trials from the switch trials. These neural switch cost maps of FC were adjusted for signal power at the whole‐brain level and were correlated with the behavioural (i.e., reaction time) switch cost in cannabis users and non‐users separately. We then compared the resulting statistical maps by group using a whole‐brain Fisher *Z* transformation [54, 55, 56, 57, 58, 59]. Coherence values adjusted for signal power were extracted from the peak voxel of the resultant significant clusters of neural activity (i.e., the voxel with the highest statistical value per cluster) per participant. To account for multiple comparisons, a significance threshold of *p* < 0.005 was used for the identification of significant clusters in all whole‐brain statistical maps, accompanied by a cluster (*k*) threshold of at least 25 contiguous voxels (i.e., 1600 mm^3^ of brain tissue) based on the theory of Gaussian random fields [60, 61, 62]. Additionally, we did not consider coherence within 4 cm of the seed region in further analyses to account for signal leakage [63]. All whole‐brain statistical analyses were computed using a custom function in MATLAB (MathWorks; Natick, Massachusetts) and other statistical analyses were conducted in IBM SPSS v.25.

### 2.10 Availability of Data

The data used in this article will be made publicly available through the COINS framework upon the completion of the study (https://coins.trendscenter.org/).

## 3 Results

### 3.1 Participant Characteristics

Of the 55 participants (25 cannabis users, 30 non‐users), eight participants (three cannabis users, five non‐users) were lost to follow‐up, one participant was excluded for low task accuracy (< 60% of trials correct), and one participant did not successfully complete the MEG scan. The final sample included 45 participants (20 cannabis users, 25 non‐users) between the ages of 20 and 60 years old (*M* = 41.28, *SD* = 10.93) who successfully completed the neuropsychological assessment, substance use interview, and the task‐switch paradigm during the MEG session. For a more detailed breakdown of demographic data for the final sample, refer to Table 1.

**TABLE 1:** Means and standard deviations of demographic measures.

	Cannabis users (*n* = 20)	Non‐users (*n* = 25)	*p*
Age	39.90 (10.07)	41.96 (10.70)	0.67
Sex (% male)	57%	60%	0.54
CUDIT‐R total score	5.76 (7.36)	—	—
Alcohol use disorder (%)	20%	8%	0.38
Learning Z‐score	0.13 (0.70)	0.56 (0.86)	0.07
Memory Z‐score	0.15 (0.63)	0.53 (0.83)	0.09
Processing speed Z‐score	0.50 (0.81)	0.40 (0.89)	0.69
Attention Z‐score	0.20 (0.77)	0.32 (0.69)	0.56
Executive function Z‐score	0.85 (0.85)	0.61 (0.79)	0.33
Language Z‐score	0.34 (0.65)	−0.07 (0.86)	0.08
Motor dexterity Z‐score	0.10 (0.75)	0.21 (0.72)	0.61
BDI‐II total score	7.10 (5.32)	4.04 (6.16)	0.09

*Note:* Percentages shown for sex and alcohol use disorder (*χ* ^*2*^ for sex and Fisher's exact test for alcohol use disorder).

### 3.2 Task‐Switch Task Performance

To assess the impact of cannabis use on behavioural performance, we conducted a 2 × 2 ANOVA with a within‐groups effect of condition (i.e., switch vs. no‐switch), and a between‐groups effect of cannabis use (i.e., cannabis users vs. non‐users). We found there was a significant main effect of condition (*F* = 49.98, *p* < 0.001), indicating that regardless of cannabis use status, participants responded more slowly on switch trials relative to no‐switch trials (i.e., the behavioural switch cost; Figure 1B). Neither the main effect of cannabis use (*F* = 0.02, *p* = 0.878) nor the cannabis use‐by‐condition interaction (*F* = 0.51, *p* = 0.477) were significant. Regarding accuracy, there were no significant main effects by group (*F* = 0.01, *p* = 0.945), condition (*F* = 0.14, *p* = 0.706), or in the group‐by‐condition interaction (*F* = 1.18, *p* = 0.283; Figure 1B).

### 3.3 Neural Oscillatory Responses

**FIGURE 2:**
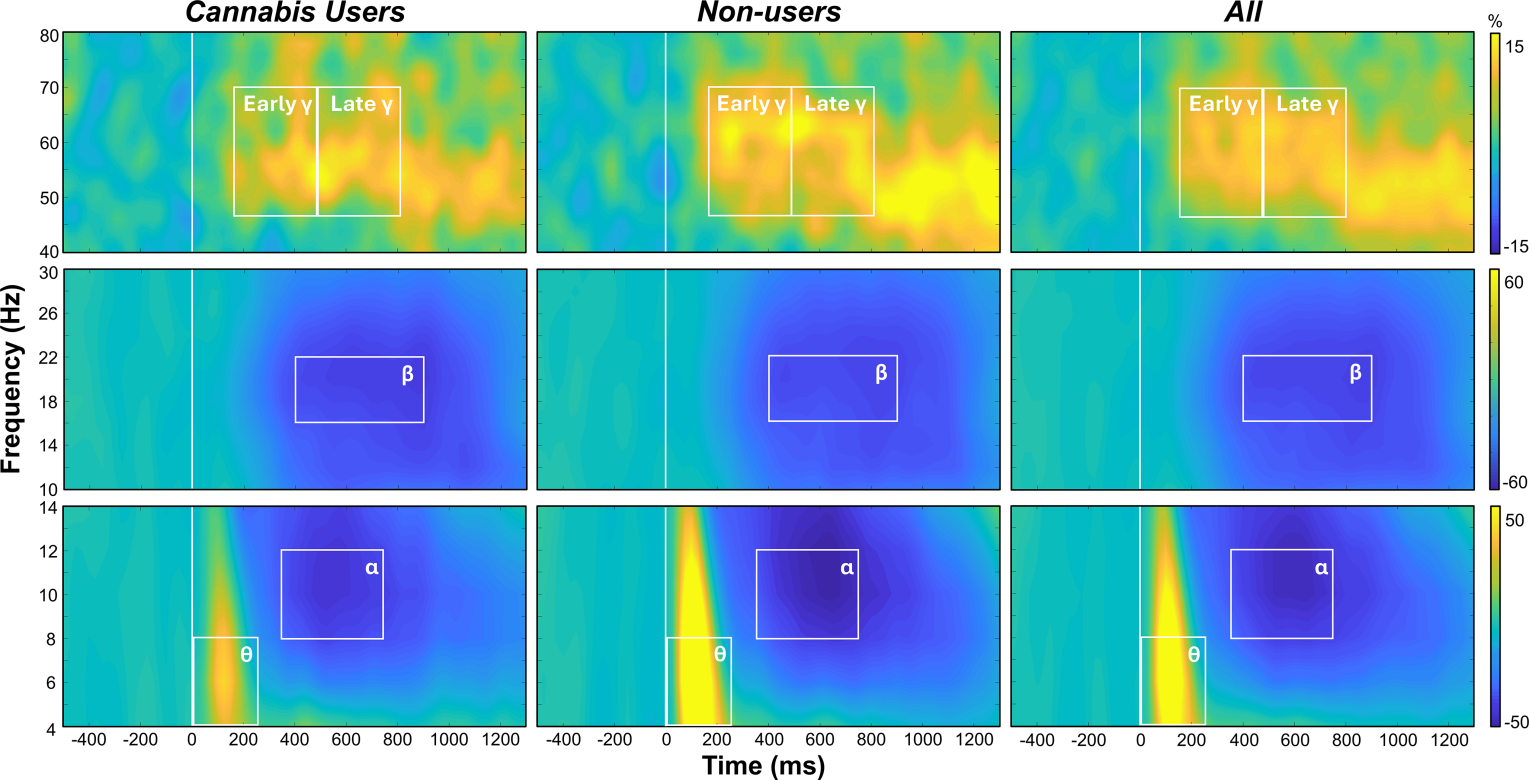
Neural responses to the task‐switch paradigm. Grand‐averaged time‐frequency spectrograms of MEG sensors illustrating significant responses: *theta* (θ; 4–8 Hz, 0–250 ms), *alpha* (α; 8–12 Hz, 350–750 ms), *beta* (β; 16–22 Hz, 400–900 ms), *early gamma* (γ; 46–70 Hz, 150–475 ms) and *late gamma* (46–70 Hz, 475–800 ms) activity. Theta and alpha responses are depicted by a parieto‐occipital sensor (MEG1923), beta responses are depicted using a parietal sensor (MEG0422), and gamma is depicted by a parieto‐occipital sensor (MEG2042). Responses are shown across all participants (*right*) and separately per group (*left and middle*). Frequencies (Hz) are shown on the *y*‐axis and time (ms) on the *x*‐axis. Signal power data are expressed as a percent difference from the baseline period with the colour scale shown with scales to the far right.

**FIGURE 3:**
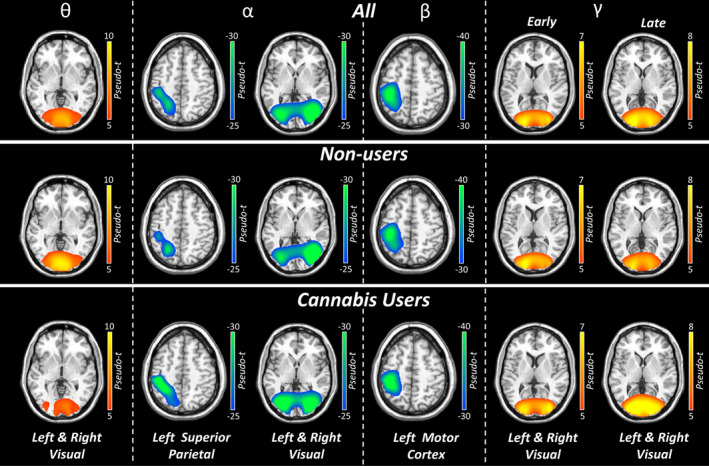
Grand‐averaged beamformer images across both conditions. Grand‐averaged beamformer maps for each oscillatory response window are shown in pseudo‐*t* units. In the top row, images have been averaged across both conditions and both groups and depict the strongest neural oscillatory responses contributing to the sensor‐level data. In the bottom two rows, images have been averaged across both conditions but are shown separately per group.

Sensor‐level analyses collapsed across both conditions and groups revealed five distinct time‐frequency windows in all participants. Specifically, significant increases in power relative to the baseline period were observed in the theta range (4–8 Hz) from 0 to 250 ms and in the gamma band (46–70 Hz) during an early (150–475 ms) and later time window (475–800 ms; Figure 2; *p*s < 0.005, corrected). In addition, decreases in power relative to the baseline period were observed in the alpha range (8–12 Hz) from 350 to 750 ms and in the beta (16–22 Hz) band from 400 to 900 ms (Figure 2; *p*s < 0.005, corrected). These windows were imaged both combined and separately across conditions (i.e., *switch* and *no‐switch*) per participant. Whole‐brain responses for the separate and combined conditions were averaged across all participants (Figure 3), which revealed bilateral theta, alpha, early and late gamma activity in the visual cortices, as well as alpha activity in the left superior parietal. The beta oscillations were centred on the left precentral gyrus (i.e., primary motor cortex) and thus were not further examined as the study focused on the cognitive features of the task and not motor performance. Average beamformer maps per condition and group are shown in Figure S1.

### 3.4 Altered Relationship Between Neural and Behavioural Switch Costs

We computed whole‐brain voxel‐wise coherence maps per condition using the DICS approach [51], with the peak voxels from the grand‐average maps across both conditions and groups being the seeds for each time‐frequency window. Next, we computed neural switch cost coherence maps (*switch* – *no‐switch*) per participant. We then ran whole‐brain correlations using the neural coherence switch cost maps and the behavioural (i.e., reaction time) switch cost for cannabis users and non‐users separately. These whole‐brain correlation maps by group are shown in Figure S2. Notably, to ensure that the amplitude of the neural response at the seed and source locations were not driving the connectivity, a voxel‐wise amplitude covariate of no interest was included in the computation. The whole‐brain correlation maps were then subjected to Fisher *r*‐to‐*z* transformations to probe for group differences in the association between FC and the behavioural switch cost using a threshold of *p* < 0.005, *k* ≥ 25.

**FIGURE 4:**
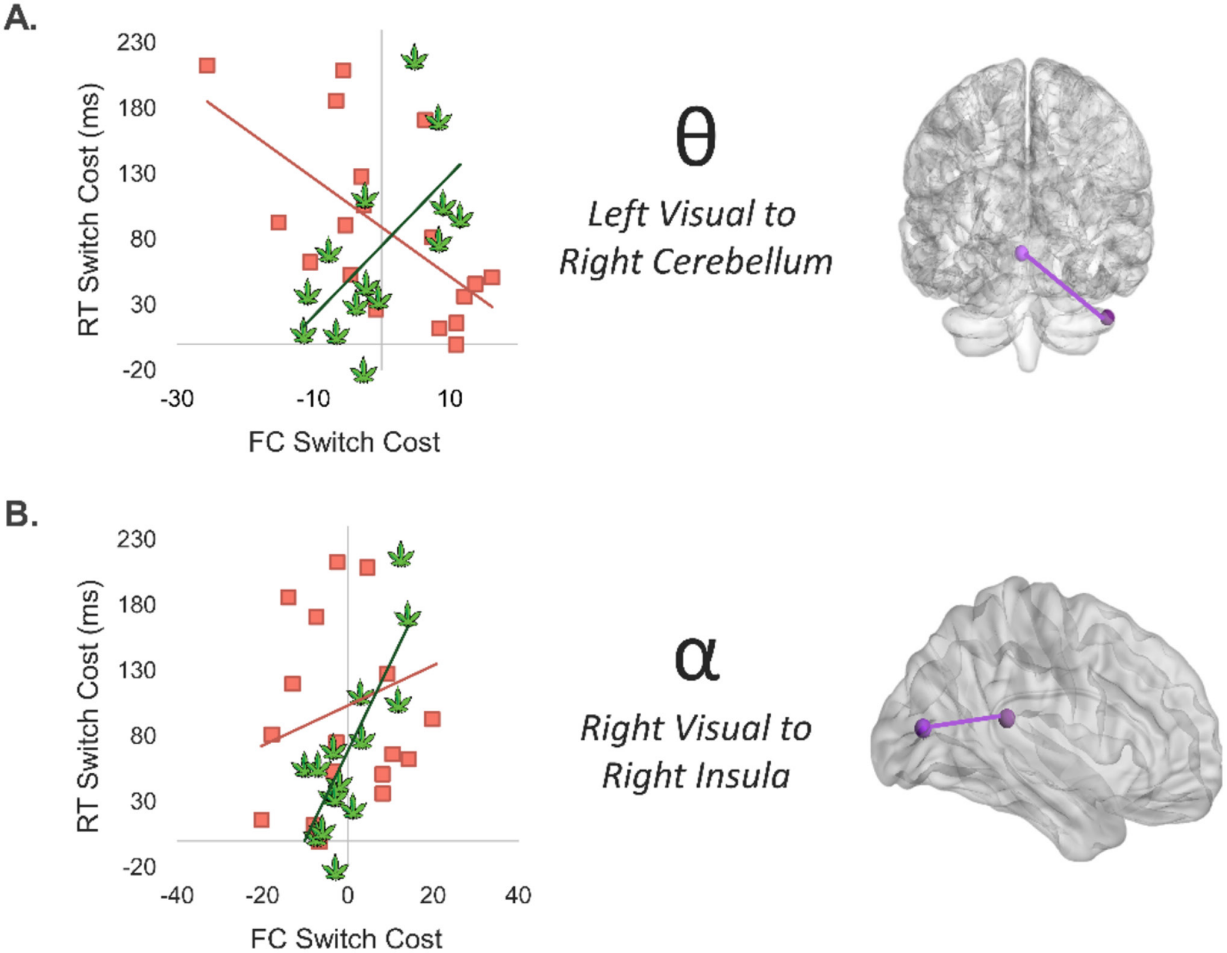
Group differences in the relationship between theta and alpha functional connectivity (FC) and behaviour during task switching. (A) Theta FC switch costs between left visual cortex and the right cerebellum were differentially associated with behavioural reaction time (RT) switch costs in users (green) compared to non‐users (salmon). Corresponding brain regions are depicted in the glass brain (right). (B) The relationship between alpha FC switch cost and RT switch costs also differed by group between right visual cortex and right insula. All Fisher *Z* scores were significant (*p* < 0.005).

Results revealed differences in the theta, alpha, early gamma, and late gamma bands. Specifically, there were differential associations in theta FC and behavioural switch costs between the left visual cortex and the right cerebellum (*r*_Non‐users_ = −0.62, *p* = 0.006; *r*_Users_ = 0.63, *p* = 0.017; Z = −3.66, *p* < 0.001; Figure 4A) and in early alpha between the right visual cortex and the right insula (*r*_Non‐users_ = 0.24, *p* = 0.341; *r*_Users_ = 0.80, *p* < 0.001; Z = −2.22, *p* = 0.026; Figure 4B).

**FIGURE 5:**
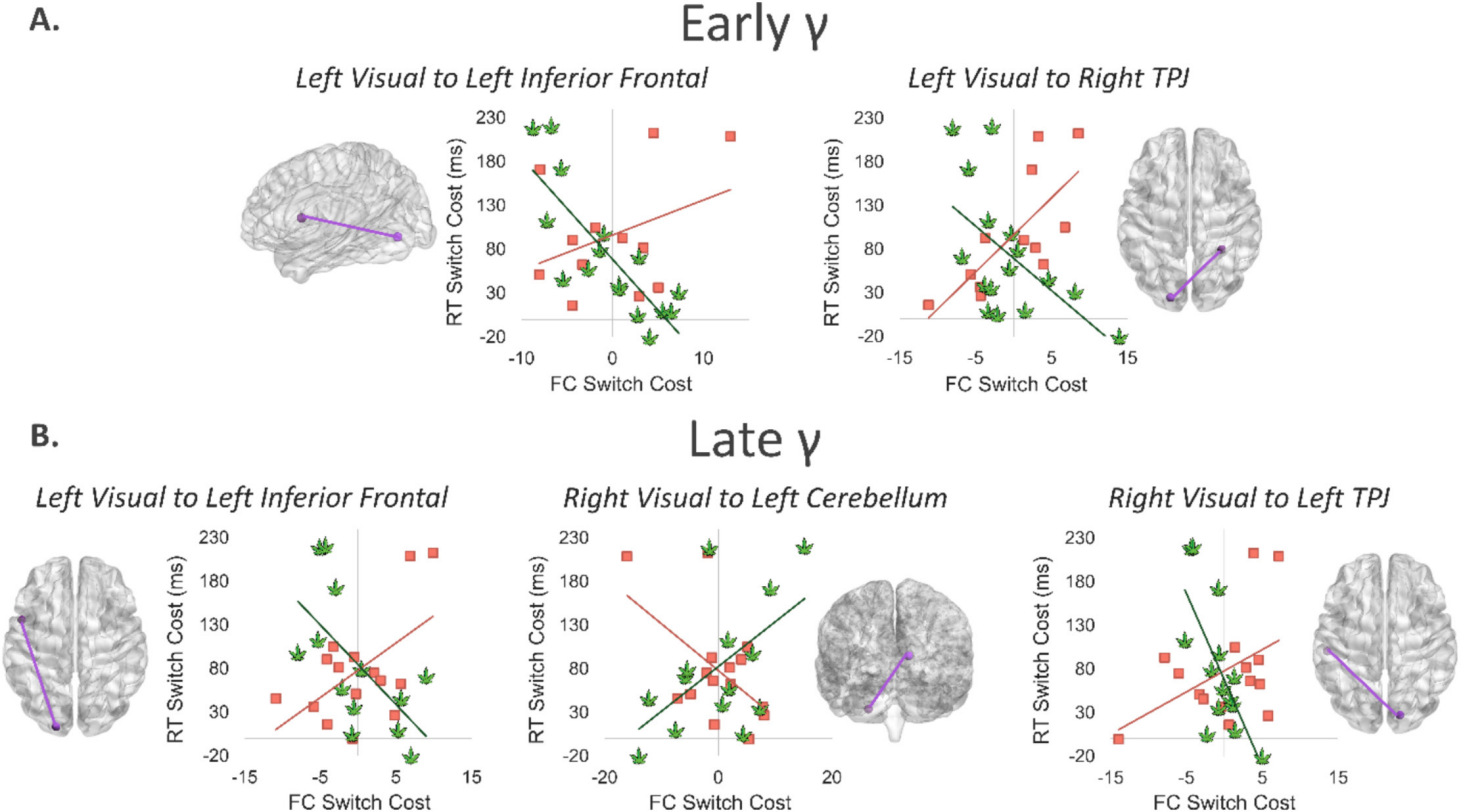
Group differences in the relationship between gamma functional connectivity and behaviour during task switching. (A) The relationship between early gamma functional connectivity (FC) switch cost and reaction time (RT) switch cost differed by group between the left visual cortex and left inferior frontal gyrus. Corresponding brain regions are depicted in the glass brain. Early gamma FC switch cost and RT switch cost association also differed by group between the left visual cortex and right temporoparietal junction (TPJ). (B) The relationship between late gamma FC switch cost and RT switch cost differed by group between the left visual cortex and left inferior frontal, as well as the right visual cortex and left cerebellum, and the right visual cortex and left TPJ. All Fisher *Z* scores were significant (*p* < 0.005).

Differences in the relationship between early gamma FC switch cost and the behavioural switch cost were found between the left visual cortex and the left inferior frontal gyrus (*r*_Non‐users_ = 0.36, *p* = 0.246; *r*_Users_ = −0.81, *p* < 0.001; Z = 3.49, *p* < 0.001; Figure 5A), as well as the right temporoparietal junction (TPJ; *r*_Non‐users_ = 0.73, *p* = 0.007; *r*_Users_ = −0.57, *p* = 0.022; Z = 3.62, *p* < 0.001; Figure 5A). Regarding the late gamma band response, cannabis use was associated with different relationships between FC and behavioural switch costs in the left visual cortex to left inferior frontal gyrus pathway (*r*_Non‐users_ = 0.56, *p* = 0.032; *r*_Users_ = −0.62, *p* = 0.023; Z = 3.17, *p* = 0.002; Figure 5B), as well as connectivity between right visual cortex and the left cerebellum (*r*_Non‐users_ = −0.62, *p* = 0.006; *r*_Users_ = 0.63, *p* = 0.017; Z = −2.98, *p* = 0.003; Figure 5B) and the left TPJ (*r*_Non‐users_ = 0.46, *p* = 0.085; *r*_Users_ = −0.71, *p* = 0.004; Z = 3.32, *p* < 0.001; Figure 5B).

## 4 Discussion

Our results indicated differential associations between FC switch costs and behavioural (i.e., reaction time) switch costs in the theta, alpha, early gamma, and late gamma bands between chronic cannabis users and non‐users. Specifically, connectivity between the visual cortices and brain regions such as the TPJ, insula, inferior frontal gyrus, and cerebellum were differently related to the behavioural switch cost effect in users, who importantly were not acutely under the influence of cannabis at the time of the MEG scan. Because cannabis users and non‐users did not significantly differ in task accuracy and reaction time, the results suggest that cannabis users may rely on altered patterns of functional connectivity within the ventral attention network to maintain adequate performance. Overall, our results are in line with our primary hypothesis that the oscillatory dynamics serving cognitive flexibility are altered in chronic cannabis users.

Theta FC between the left visual cortex and the right cerebellum during task switching sharply increased as a function of the behavioural switch cost in cannabis users. Theta oscillations are known to be important for the temporal organization of incoming visual stimuli, and theta activity in the occipital cortices has been strongly tied to visual processing and initial sensory encoding [64, 65, 66, 67]. Theta band oscillatory activity and FC in the fronto‐parietal network are known to be crucial for supporting the top‐down orientation of attention and cognitive control processing [14, 68, 69, 70, 71], and it has been shown to be aberrant in cannabis users during tasks that probe higher‐order cognition, as noted previously [17, 18]. These documented alterations in theta activity further suggest compensatory processing among chronic cannabis users.

Our theta findings in the cerebellum are particularly noteworthy, as even though CB_1_ receptors are located throughout the brain, the cerebellum has a high concentration of these receptors [72]. In fact, resting‐state fMRI studies have reported cannabis‐related alterations to cerebellar‐cortical connectivity [33, 73, 74]. Cerebellar findings also emerged in the late gamma band, as there were cross‐hemispheric FC switch costs from the right visual cortex to the left cerebellum. These findings were in the same direction as the theta FC results in regard to group effects and brain regions involved, but the laterality pattern was reversed. These results further underscore the overall importance of the cerebellum in task switching and the impact that cannabis use has on this region. Interestingly, beyond this specific pathway, larger FC switch costs were associated with smaller behavioural switch costs in the early and late gamma bands in cannabis users. Gamma activity is especially important for attention, with stronger gamma activity being associated with the bottom‐up attentional processing within the VAN [75]. Further, it has been suggested that oscillatory gamma activity supports stronger coupling between sensory regions, namely, the visual and frontal cortices during attention tasks [75]. As mentioned previously, cannabis has been shown to alter gamma activity via modulation of CB_1_ receptors [26]. In the cortex, these presynaptic receptors inhibit the release of γ‐aminobutyric acid (GABA) from interneurons, which are theorized to be the driving force of gamma oscillatory activity [76, 77, 78]. Due to the presence of CB_1_ receptors throughout the cortex and cerebellum, it follows that the majority of our cannabis findings are in the gamma frequency band, which is purported to be the most heavily impacted by endocannabinoid receptor activation.

In addition to differences in the theta and gamma bands, we found FC effects in the alpha range during task switching. Neural oscillatory activity in the alpha (and gamma) spectral windows is consistent with a previous MEG study of cognitive flexibility among non‐users, which also found involvement of the VAN [14]. The VAN is important for detecting and orienting attention to relevant stimuli by supporting bottom‐up processing and involves brain regions such as the TPJ, ventral frontal cortex, inferior frontal gyrus, and insula [12, 70, 79, 80, 81]. As the VAN is critical for task switching, it follows that in the present study, stronger connectivity between visual cortices and regions of the VAN were associated with reduced behavioural switch costs in cannabis users. This was also true for pathways involving the TPJ in the early and late gamma frequencies, which is a region of the VAN that supports the detection of salient stimuli and sustained attention [80]. In another MEG study regarding chronic cannabis use and attentional processing, differential alpha activity was also observed in the insula, as well as the ACC, among users compared to non‐users in the absence of behavioural differences [67]. This work provides further evidence of compensatory processing among chronic users to effectively shift attention and support the behaviourally relevant control over lower‐order attention network nodes [12, 82].

It is important to contextualize these results in terms of substance use behaviour. One limitation of this study is that we focused solely on adult cannabis users, though differences in how cannabis use impacts attention and executive function among adolescents and adults have been documented [83]. Future studies should further probe the effects that cannabis use may have during the transition from adolescence to adulthood. Another limitation of our study is that our sample was composed of chronic heavy cannabis users that had been using cannabis regularly for at least the past three years, so these results may not generalize to infrequent cannabis users. It is also crucial to emphasize that these findings reflect the impact of regular long‐term cannabis use rather than the acute effects of cannabis, and thus, our findings would likely differ from studies focusing on the acute effects of cannabis use. Another possible limitation surrounds our task design, as it is possible that we did not find differences in task performance between groups due to the task not being sufficiently challenging to elicit cognitive flexibility deficits in the users. A more difficult task in this domain may result in significant behavioural differences between users and non‐users, and this should be a priority in future studies. Also, we did not collect information regarding recent caffeine use that could impact task performance, and such data should be collected in future work. Finally, it is important to acknowledge that other FC methods such as the phase locking value, which looks at the phase relationships between two cortical sources over time, may yield different patterns of connectivity than the coherence measures used in the present study. Using alternative methods for computing FC and taking a dynamic FC approach [84, 85, 86, 87, 88, 89] will be important in future work and should be a priority.

In summary, this study provides evidence that chronic cannabis users rely on different patterns of FC to minimize costs during task switching. This is in line with previous literature indicating altered neural dynamics underlying attention and attention reorientation in cannabis users compared to non‐users, despite similar task performance [16, 17, 18]. We conclude that our results are indicative of altered compensatory processes within the VAN in cannabis users to maintain adequate task performance.

## Supporting information


**Figure S1.** Average beamformer maps per condition and group.
**Figure S2.** Whole‐brain correlation maps.

## Data Availability

The data used in this article will be made publicly available through the COINS framework upon the completion of the study (https://coins.trendscenter.org/).

## References

[1] M. E. Patrick , R. A. Miech , L. D. Johnston , and P. M. O'Malley , “Monitoring the Future Panel Study Annual Report: National Data on Substance use Among Adults Ages 19 to 60, 1976–2022,” (2023), Monitoring the Future Monograph Series. Institute for Social Research, University of Michigan, 10.7826/ISR-UM.06.585140.002.07.0002.2023.

[2] M. E. Patrick , J. E. Schulenberg , R. A. Miech , L. D. Johnston , P. M. O'Malley , and J. G. Bachman , “Monitoring the Future Panel Study Annual Report: National Data on Substance Use Among Adults Ages 19 to 60, 1976–2021,” Monitoring the Future Monograph Series. Institute for Social Research, University of Michigan, (2022), 10.7826/ISR-UM.06.585140.002.07.0001.2022.

[3] A. Batalla , S. Bhattacharyya , M. Yücel , et al., “Structural and Functional Imaging Studies in Chronic Cannabis Users: A Systematic Review of Adolescent and Adult Findings,” PLoS ONE 8, no. 2 (2013): e55821, 10.1371/journal.pone.0055821. 23390554 PMC3563634

[4] M. E. Lovell , J. Akhurst , C. Padgett , M. I. Garry , and A. Matthews , “Cognitive Outcomes Associated With Long‐Term, Regular, Recreational Cannabis Use in Adults: A Meta‐Analysis,” Experimental and Clinical Psychopharmacology 28 (2020): 471–494, 10.1037/pha0000326. 31670548

[5] K. A. Sagar , M. K. Dahlgren , A. Gönenç , M. T. Racine , M. W. Dreman , and S. A. Gruber , “The Impact of Initiation: Early Onset Marijuana Smokers Demonstrate Altered Stroop Performance and Brain Activation,” Developmental Cognitive Neuroscience 16 (2015): 84–92, 10.1016/j.dcn.2015.03.003. 25936584 PMC4596753

[6] N. Solowij , R. S. Stephens , R. A. Roffman , et al., “Cognitive Functioning of Long‐Term Heavy cannabis Users Seeking Treatment,” JAMA 287, no. 9 (2002): 1123–1131, 10.1001/jama.287.9.1123. 11879109

[7] N. D. Volkow , R. D. Baler , W. M. Compton , and S. R. B. Weiss , “Adverse Health Effects of Marijuana Use,” New England Journal of Medicine 370, no. 23 (2014): 2219–2227, 10.1056/nejmra1402309. 24897085 PMC4827335

[8] R. D. Crean , N. A. Crane , and B. J. Mason , “An Evidence Based Review of Acute and Long‐Term Effects of Cannabis Use on Executive Cognitive Functions,” Journal of Addiction Medicine 5, no. 1 (2011): 1–8, 10.1097/ADM.0b013e31820c23fa. 21321675 PMC3037578

[9] L. Dellazizzo , S. Potvin , S. Giguère , and A. Dumais , “Evidence on the Acute and Residual Neurocognitive Effects of Cannabis Use in Adolescents and Adults: A Systematic Meta‐Review of Meta‐Analyses,” Addiction (Abingdon, England) 117, no. 7 (2022): 1857–1870, 10.1111/add.15764. 35048456

[10] J. C. Duperrouzel , K. Granja , I. Pacheco‐Colón , and R. Gonzalez , “Adverse Effects of Cannabis Use on Neurocognitive Functioning: A Systematic Review of Meta‐Analytic Studies,” Journal of Dual Diagnosis 16, no. 1 (2020): 43–57, 10.1080/15504263.2019.1626030. 31232216 PMC6925658

[11] A. M. Schreiner and M. E. Dunn , “Residual Effects of Cannabis Use on Neurocognitive Performance After Prolonged Abstinence: A Meta‐Analysis,” Experimental and Clinical Psychopharmacology 20, no. 5 (2012): 420–429, 10.1037/a0029117. 22731735

[12] D. R. Dajani and L. Q. Uddin , “Demystifying Cognitive Flexibility: Implications for Clinical and Developmental Neuroscience,” Trends in Neurosciences 38, no. 9 (2015): 571–578, 10.1016/j.tins.2015.07.003. 26343956 PMC5414037

[13] M. W. Cole , J. R. Reynolds , J. D. Power , G. Repovs , A. Anticevic , and T. S. Braver , “Multi‐Task Connectivity Reveals Flexible Hubs for Adaptive Task Control,” Nature Neuroscience 16, no. 9 (2013): 1348–1355, 10.1038/nn.3470. 23892552 PMC3758404

[14] A. L. Proskovec , A. I. Wiesman , and T. W. Wilson , “The Strength of Alpha and Gamma Oscillations Predicts Behavioral Switch Costs,” NeuroImage 188 (2019): 274–281, 10.1016/j.neuroimage.2018.12.016. 30543844 PMC6401274

[15] L. Qiao , M. Xu , X. Luo , L. Zhang , H. Li , and A. Chen , “Flexible Adjustment of the Effective Connectivity Between the Fronto‐Parietal and Visual Regions Supports Cognitive Flexibility,” NeuroImage 220 (2020): 117158, 10.1016/j.neuroimage.2020.117158. 32659352

[16] K. M. McDonald , M. Schantell , L. K. Horne , et al., “The Neural Oscillations Serving Task Switching Are Altered in Cannabis Users,” Journal of Psychopharmacology 38 (2024): 471–480, 10.1177/02698811241235204. 38418434 PMC11488983

[17] S. D. Springer , R. K. Spooner , M. Schantell , et al., “Regular Recreational Cannabis Users Exhibit Altered Neural Oscillatory Dynamics During Attention Reorientation,” Psychological Medicine 53, no. 4 (2021): 1–10, 10.1017/S0033291721002671. 34889178 PMC9250753

[18] A. Rangel‐Pacheco , B. J. Lew , M. D. Schantell , et al., “Altered Fronto‐Occipital Connectivity During Visual Selective Attention in Regular Cannabis Users,” Psychopharmacology 238, no. 5 (2021): 1351–1361, 10.1007/s00213-020-05717-3. 33241479 PMC8068572

[19] M. Hajós , W. E. Hoffmann , and B. Kocsis , “Activation of Cannabinoid‐1 Receptors Disrupts Sensory Gating and Neuronal Oscillation: Relevance to Schizophrenia,” Biological Psychiatry 63, no. 11 (2008): 1075–1083, 10.1016/j.biopsych.2007.12.005. 18261715

[20] I. Katona , B. Sperlágh , A. Sík , et al., “Presynaptically Located CB1 Cannabinoid Receptors Regulate GABA Release From Axon Terminals of Specific Hippocampal Interneurons,” Journal of Neuroscience: The Official Journal of the Society for Neuroscience 19, no. 11 (1999): 4544–4558, 10.1523/JNEUROSCI.19-11-04544.1999. 10341254 PMC6782612

[21] N. H. Morgan , I. M. Stanford , and G. L. Woodhall , “Modulation of Network Oscillatory Activity and GABAergic Synaptic Transmission by CB1 Cannabinoid Receptors in the Rat Medial Entorhinal Cortex,” Neural Plasticity 2008 (2008): 808564, 10.1155/2008/808564. 19079598 PMC2593022

[22] M. Glass , M. Dragunow , and R. L. Faull , “Cannabinoid Receptors in the Human Brain: A Detailed Anatomical and Quantitative Autoradiographic Study in the Fetal, Neonatal and Adult Human Brain,” Neuroscience 77, no. 2 (1997): 299–318, 10.1016/s0306-4522(96)00428-9. 9472392

[23] K. Mackie , “Distribution of Cannabinoid Receptors in the Central and Peripheral Nervous System,” Handbook of Experimental Pharmacology 168 (2005): 299–325, 10.1007/3-540-26573-2_10. 16596779

[24] B. Bietar , S. Tanner , and C. Lehmann , “Neuroprotection and Beyond: The Central Role of CB1 and CB2 Receptors in Stroke Recovery,” International Journal of Molecular Sciences 24, no. 23 (2023): 16728, 10.3390/ijms242316728. 38069049 PMC10705908

[25] P. D. Skosnik , M. Hajós , J. A. Cortes‐Briones , et al., “Cannabinoid Receptor‐Mediated Disruption of Sensory Gating and Neural Oscillations: A Translational Study in Rats and Humans,” Neuropharmacology 135 (2018): 412–423, 10.1016/j.neuropharm.2018.03.036. 29604295 PMC6091633

[26] P. D. Skosnik , G. P. Krishnan , D. C. D'Souza , W. P. Hetrick , and B. F. O'Donnell , “Disrupted Gamma‐Band Neural Oscillations During Coherent Motion Perception in Heavy Cannabis Users,” Neuropsychopharmacology: Official Publication of the American College of Neuropsychopharmacology 39, no. 13 (2014): 3087–3099, 10.1038/npp.2014.166. 24990428 PMC4229582

[27] A. D. Thames , N. Arbid , and P. Sayegh , “Cannabis Use and Neurocognitive Functioning in a Non‐Clinical Sample of Users,” Addictive Behaviors 39, no. 5 (2014): 994–999, 10.1016/j.addbeh.2014.01.019. 24556155 PMC4032061

[28] Y. Arif , A. I. Wiesman , N. J. Christopher‐Hayes , and T. W. Wilson , “Aberrant Inhibitory Processing in the Somatosensory Cortices of Cannabis‐Users,” Journal of Psychopharmacology (Oxford, England) 35, no. 11 (2021): 1356–1364, 10.1177/02698811211050557. 34694190 PMC9659470

[29] C. A. Castelblanco , S. D. Springer , M. Schantell , et al., “Chronic Cannabis Users Exhibit Altered Oscillatory Dynamics and Functional Connectivity Serving Visuospatial Processing,” Journal of Psychopharmacology (Oxford, England) 38, no. 8 (2024): 724–734, 10.1177/02698811241265764. 39087306 PMC11471968

[30] M. Schantell , J. A. John , A. T. Coutant , et al., “Chronic cannabis use Alters the Spontaneous and Oscillatory Gamma Dynamics Serving Cognitive Control,” Human Brain Mapping 45, no. 11 (2024): e26787, 10.1002/hbm.26787. 39023178 PMC11256138

[31] L. K. Webert , M. Schantell , J. A. John , et al., “Regular Cannabis Use Modulates Gamma Activity in Brain Regions Serving Motor Control,” Journal of Psychopharmacology (Oxford, England) 38, no. 11 (2024): 949–960, 10.1177/02698811241268876. 39140179 PMC11524774

[32] L. Chang , R. Yakupov , C. Cloak , and T. Ernst , “Marijuana Use Is Associated With a Reorganized Visual‐Attention Network and Cerebellar Hypoactivation,” Brain: A Journal of Neurology 129, no. Pt 5 (2006): 1096–1112, 10.1093/brain/awl064. 16585053

[33] A. M. Schnakenberg Martin , D. J. Kim , S. D. Newman , et al., “Altered Cerebellar‐Cortical Resting‐State Functional Connectivity in Cannabis Users,” Journal of Psychopharmacology (Oxford, England) 35, no. 7 (2021): 823–832, 10.1177/02698811211019291. 34034553 PMC8813046

[34] R. R. Wetherill , Z. Fang , K. Jagannathan , A. R. Childress , H. Rao , and T. R. Franklin , “Cannabis, Cigarettes, and Their Co‐occurring use: Disentangling Differences in Default Mode Network Functional Connectivity,” Drug and Alcohol Dependence 153 (2015): 116–123. 26094186 10.1016/j.drugalcdep.2015.05.046 PMC4509835

[35] I. H. Harding , N. Solowij , B. J. Harrison , et al., “Functional Connectivity in Brain Networks Underlying Cognitive Control in Chronic Cannabis Users,” Neuropsychopharmacology: Official Publication of the American College of Neuropsychopharmacology 37, no. 8 (2012): 1923–1933, 10.1038/npp.2012.39. 22534625 PMC3376324

[36] D. Wechsler , Wechsler Memory Scale – Third Edition (San Antonio: Psychological Corporation, 1997a).

[37] D. C. Delis , J. H. Kramer , E. Kaplan , and B. A. Ober , The California Verbal Learning Test – Second Edition (San Antonio: Psychological Corporation, 2000).

[38] P. E. Comalli , S. Wapner , and H. Werner , “Interference Effects of Stroop Color‐Word Test in Childhood, Adulthood, and Aging,” Journal of Genetic Psychology 100, no. 1 (1962): 47–53, 10.1080/00221325.1962.10533572. 13880724

[39] R. K. Heaton , “Revised Comprehensive Norms for an Expanded Halstead‐Reitan Battery: Demographically Adjusted Neuropsychological Norms for African American and Caucasian Adults, Professional Manual,” Psychological Assessment Resources, (2004).

[40] D. Wechsler , Wechsler Adult Intelligence Scale – Third Edition (San Antonio: Psychological Corporation, 1997b).

[41] H. Kløve , “Clinical Neuropsychology,” Medical Clinics of North America 47, no. 6 (1963): 1647–1658, 10.1016/S0025-7125(16)33515-5. 14078168

[42] G. Wilkinson and G. Robertson , Wide Range Achievement Test 4 Professional Manual (Lutz, FL: Psychological Assessment Resources, 2006).

[43] A. T. Beck , R. A. Steer , and G. Brown , “Beck Depression Inventory–II (BDI‐II) [Database Record],” PsycTESTS, (1996), 10.1037/t00742-000.

[44] A. I. Wiesman , N. J. Christopher‐Hayes , and T. W. Wilson , “Stairway to Memory: Left‐Hemispheric Alpha Dynamics Index the Progressive Loading of Items Into a Short‐Term Store,” NeuroImage 235 (2021): 118024, 10.1016/j.neuroimage.2021.118024. 33836267 PMC8354033

[45] A. I. Wiesman and T. W. Wilson , “Attention Modulates the Gating of Primary Somatosensory Oscillations,” NeuroImage 211 (2020): 116610, 10.1016/j.neuroimage.2020.116610. 32044438 PMC7111587

[46] S. Taulu and J. Simola , “Spatiotemporal Signal Space Separation Method for Rejecting Nearby Interference in MEG Measurements,” Physics in Medicine and Biology 51, no. 7 (2006): 1759–1768, 10.1088/0031-9155/51/7/008. 16552102

[47] M. A. Uusitalo and R. J. Ilmoniemi , “Signal‐Space Projection Method for Separating MEG or EEG Into Components,” Medical & Biological Engineering & Computing 35, no. 2 (1997): 135–140, 10.1007/BF02534144. 9136207

[48] C. K. Kovach and P. E. Gander , “The Demodulated Band Transform,” Journal of Neuroscience Methods 261 (2016): 135–154, 10.1016/j.jneumeth.2015.12.004. 26711370 PMC5084918

[49] N. Papp and P. Ktonas , “Critical Evaluation of Complex Demodulation Techniques for the Quantification of Bioelectrical Activity,” Biomedical Sciences Instrumentation 13 (1977): 135–145. 871500

[50] S. S. Dalal , K. Sekihara , and S. S. Nagarajan , “Modified Beamformers for Coherent Source Region Suppression,” IEEE Transactions on Biomedical Engineering 53, no. 7 (2006): 1357–1363, 10.1109/TBME.2006.873752. 16830939 PMC3066091

[51] J. Gross , J. Kujala , M. Hämäläinen , L. Timmermann , A. Schnitzler , and R. Salmelin , “Dynamic Imaging of Coherent Sources: Studying Neural Interactions in the Human Brain,” Proceedings of the National Academy of Sciences 98, no. 2 (2001): 694–699, 10.1073/pnas.98.2.694. PMC14650 11209067

[52] B. D. Van Veen , W. Van Drongelen , M. Yuchtman , and A. Suzuki , “Localization of Brain Electrical Activity via Linearly Constrained Minimum Variance Spatial Filtering,” IEEE Transactions on Biomedical Engineering 44, no. 9 (1997): 867–880, 10.1109/10.623056. 9282479

[53] A. Hillebrand , K. D. Singh , I. E. Holliday , P. L. Furlong , and G. R. Barnes , “A New Approach to Neuroimaging With Magnetoencephalography,” Human Brain Mapping 25, no. 2 (2005): 199–211, 10.1002/hbm.20102. 15846771 PMC6871673

[54] C. M. Embury , A. I. Wiesman , A. L. Proskovec , et al., “Neural Dynamics of Verbal Working Memory Processing in Children and Adolescents,” NeuroImage 185 (2019): 191–197, 10.1016/j.neuroimage.2018.10.038. 30336254 PMC6289659

[55] M. H. Fung , R. L. Rahman , B. K. Taylor , et al., “The Impact of Pubertal DHEA on the Development of Visuospatial Oscillatory Dynamics,” Human Brain Mapping 43, no. 17 (2022): 5154–5166, 10.1002/hbm.25991. 35778797 PMC9812248

[56] M. H. Fung , B. K. Taylor , B. J. Lew , et al., “Sexually Dimorphic Development in the Cortical Oscillatory Dynamics Serving Early Visual Processing,” Developmental Cognitive Neuroscience 50 (2021): 100968, 10.1016/j.dcn.2021.100968. 34102602 PMC8187257

[57] B. R. Groff , A. I. Wiesman , M. T. Rezich , et al., “Age‐Related Visual Dynamics in HIV‐Infected Adults With Cognitive Impairment,” Neurology ‐ Neuroimmunology Neuroinflammation 7, no. 3 (2020): e690, 10.1212/NXI.0000000000000690. 32102916 PMC7051212

[58] H. Hotelling , “New Light on the Correlation Coefficient and Its Transforms,” Journal of the Royal Statistical Society: Series B: Methodological 15, no. 2 (1953): 193–232.

[59] B. K. Taylor , J. A. Eastman , M. R. Frenzel , et al., “Neural Oscillations Underlying Selective Attention Follow Sexually Divergent Developmental Trajectories During Adolescence,” Developmental Cognitive Neuroscience 49 (2021): 100961, 10.1016/j.dcn.2021.100961. 33984667 PMC8131898

[60] J. B. Poline , K. J. Worsley , A. P. Holmes , R. S. J. Frackowiak , and K. J. Friston , “Estimating Smoothness in Statistical Parametric Maps: Variability of p Values,” Journal of Computer Assisted Tomography 19, no. 5 (1995): 788–796. 7560327 10.1097/00004728-199509000-00017

[61] K. J. Worsley , M. Andermann , T. Koulis , D. MacDonald , and A. C. Evans , “Detecting Changes in Nonisotropic Images,” Human Brain Mapping 8, no. 2–3 (1999): 98–101. 10524599 10.1002/(SICI)1097-0193(1999)8:2/3<98::AID-HBM5>3.0.CO;2-F PMC6873343

[62] K. J. Worsley , S. Marrett , P. Neelin , A. C. Vandal , K. J. Friston , and A. C. Evans , “A Unified Statistical Approach for Determining Significant Signals in Images of Cerebral Activation,” Human Brain Mapping 4, no. 1 (1996): 58–73. 20408186 10.1002/(SICI)1097-0193(1996)4:1<58::AID-HBM4>3.0.CO;2-O

[63] M. J. Brookes , J. R. Hale , J. M. Zumer , et al., “Measuring Functional Connectivity Using MEG: Methodology and Comparison With fcMRI,” NeuroImage 56, no. 3 (2011): 1082–1104, 10.1016/j.neuroimage.2011.02.054. 21352925 PMC3224862

[64] Y. Arif , R. K. Spooner , A. I. Wiesman , et al., “Prefrontal Multielectrode Transcranial Direct Current Stimulation Modulates Performance and Neural Activity Serving Visuospatial Processing,” Cerebral Cortex 30, no. 9 (2020): 4847–4857, 10.1093/cercor/bhaa077. 32390042 PMC7391278

[65] P. T. Goodbourn and J. D. Forte , “Spatial Limitations of Fast Temporal Segmentation Are Best Modeled by V1 Receptive Fields,” Journal of Vision 13, no. 13 (2013): 23, 10.1167/13.13.23. 24273225

[66] A. M. Harris , P. E. Dux , C. N. Jones , and J. B. Mattingley , “Distinct Roles of Theta and Alpha Oscillations in the Involuntary Capture of Goal‐Directed Attention,” NeuroImage 152 (2017): 171–183, 10.1016/j.neuroimage.2017.03.008. 28274832

[67] A. I. Wiesman , E. Heinrichs‐Graham , A. L. Proskovec , T. J. McDermott , and T. W. Wilson , “Oscillations During Observations: Dynamic Oscillatory Networks Serving Visuospatial Attention,” Human Brain Mapping 38, no. 10 (2017): 5128–5140, 10.1002/hbm.23720. 28714584 PMC5593769

[68] P. Fries , “Rhythms for Cognition: Communication Through Coherence,” Neuron 88, no. 1 (2015): 220–235, 10.1016/j.neuron.2015.09.034. 26447583 PMC4605134

[69] T. J. McDermott , A. I. Wiesman , A. L. Proskovec , E. Heinrichs‐Graham , and T. W. Wilson , “Spatiotemporal Oscillatory Dynamics of Visual Selective Attention During a Flanker Task,” NeuroImage 156 (2017): 277–285, 10.1016/j.neuroimage.2017.05.014. 28501539 PMC5548621

[70] A. L. Proskovec , E. Heinrichs‐Graham , A. I. Wiesman , T. J. McDermott , and T. W. Wilson , “Oscillatory Dynamics in the Dorsal and Ventral Attention Networks During the Reorienting of Attention,” Human Brain Mapping 39, no. 5 (2018): 2177–2190, 10.1002/hbm.23997. 29411471 PMC5895484

[71] R. K. Spooner , A. I. Wiesman , A. L. Proskovec , E. Heinrichs‐Graham , and T. W. Wilson , “Prefrontal theta Modulates Sensorimotor Gamma Networks During the Reorienting of Attention,” Human Brain Mapping 41, no. 2 (2020): 520–529, 10.1002/hbm.24819. 31621977 PMC7268018

[72] M. Herkenham , A. B. Lynn , M. D. Little , et al., “Cannabinoid Receptor Localization in Brain,” Proceedings of the National Academy of Sciences of the United States of America 87, no. 5 (1990): 1932–1936, 10.1073/pnas.87.5.1932. 2308954 PMC53598

[73] L. E. Klumpers , D. M. Cole , N. Khalili‐Mahani , et al., “Manipulating Brain Connectivity With δ⁹‐Tetrahydrocannabinol: A Pharmacological Resting State FMRI Study,” NeuroImage 63, no. 3 (2012): 1701–1711, 10.1016/j.neuroimage.2012.07.051. 22885247

[74] C. Orr , R. Morioka , B. Behan , et al., “Altered Resting‐State Connectivity in Adolescent Cannabis Users,” American Journal of Drug and Alcohol Abuse 39, no. 6 (2013): 372–381, 10.3109/00952990.2013.848213. 24200207

[75] H. A. ElShafei , L. Fornoni , R. Masson , O. Bertrand , and A. Bidet‐Caulet , “What's in Your Gamma? Activation of the Ventral Fronto‐Parietal Attentional Network in Response to Distracting Sounds,” Cerebral Cortex 30, no. 2 (2020): 696–707, 10.1093/cercor/bhz119. 31219542

[76] A. B. Ali and M. Todorova , “Asynchronous Release of GABA via Tonic Cannabinoid Receptor Activation at Identified Interneuron Synapses in rat CA1,” European Journal of Neuroscience 31, no. 7 (2010): 1196–1207, 10.1111/j.1460-9568.2010.07165.x. 20345910

[77] G. Buzsáki and X. J. Wang , “Mechanisms of Gamma Oscillations,” Annual Review of Neuroscience 35 (2012): 203–225, 10.1146/annurev-neuro-062111-150444. PMC4049541 22443509

[78] G. Gonzalez‐Burgos and D. A. Lewis , “GABA Neurons and the Mechanisms of Network Oscillations: Implications for Understanding Cortical Dysfunction in Schizophrenia,” Schizophrenia Bulletin 34, no. 5 (2008): 944–961, 10.1093/schbul/sbn070. 18586694 PMC2518635

[79] A. M. Jimenez , J. Lee , J. K. Wynn , et al., “Abnormal Ventral and Dorsal Attention Network Activity During Single and Dual Target Detection in Schizophrenia,” Frontiers in Psychology 7 (2016): 323, 10.3389/fpsyg.2016.00323. 27014135 PMC4781842

[80] A. Kucyi , M. Hodaie , and K. D. Davis , “Lateralization in Intrinsic Functional Connectivity of the Temporoparietal Junction With Salience‐ and Attention‐Related Brain Networks,” Journal of Neurophysiology 108, no. 12 (2012): 3382–3392, 10.1152/jn.00674.2012. 23019004

[81] R. M. Umarova , D. Saur , S. Schnell , et al., “Structural Connectivity for Visuospatial Attention: Significance of Ventral Pathways,” Cerebral Cortex 20, no. 1 (2010): 121–129, 10.1093/cercor/bhp086. 19406904

[82] S. M. Doesburg , N. Bedo , and L. M. Ward , “Top‐Down Alpha Oscillatory Network Interactions During Visuospatial Attention Orienting,” NeuroImage 132 (2016): 512–519, 10.1016/j.neuroimage.2016.02.076. 26952198

[83] S. J. Broyd , H. H. van Hell , C. Beale , M. Yücel , and N. Solowij , “Acute and Chronic Effects of Cannabinoids on Human Cognition—A Systematic Review,” Biological Psychiatry 79, no. 7 (2016): 557–567, 10.1016/j.biopsych.2015.12.002. 26858214

[84] E. Heinrichs‐Graham and T. W. Wilson , “Coding Complexity in the Human Motor Circuit,” Human Brain Mapping 36, no. 12 (2015): 5155–5167, 10.1002/hbm.23000. 26406479 PMC4715608

[85] J. J. Son , Y. Arif , H. J. Okelberry , et al., “Aging Modulates the Impact of Cognitive Interference Subtypes on Dynamic Connectivity Across a Distributed Motor Network,” NPJ Aging 10, no. 1 (2024): 54, 10.1038/s41514-024-00182-0. 39580466 PMC11585575

[86] R. K. Spooner , A. I. Wiesman , and T. W. Wilson , “Peripheral Somatosensory Entrainment Modulates the Cross‐Frequency Coupling of Movement‐Related Theta‐Gamma Oscillations,” Brain Connectivity 12, no. 6 (2022): 524–537, 10.1089/brain.2021.0003. 34269624 PMC9419931

[87] R. K. Spooner and T. W. Wilson , “Cortical Theta‐Gamma Coupling Governs the Adaptive Control of Motor Commands,” Brain Communications 4, no. 6 (2022): fcac249, 10.1093/braincomms/fcac249. 36337344 PMC9631971

[88] L. Weyrich , Y. Arif , M. Schantell , et al., “Altered Functional Connectivity and Oscillatory Dynamics in Polysubstance and cannabis Only Users During Visuospatial Processing,” Psychopharmacology 240, no. 4 (2023): 769–783, 10.1007/s00213-023-06318-6. 36752815 PMC10545949

[89] A. I. Wiesman , E. Heinrichs‐Graham , T. J. McDermott , P. M. Santamaria , H. E. Gendelman , and T. W. Wilson , “Quiet Connections: Reduced Fronto‐Temporal Connectivity in Nondemented Parkinson's Disease During Working Memory Encoding,” Human Brain Mapping 37, no. 9 (2016): 3224–3235, 10.1002/hbm.23237. 27151624 PMC4980162

