# Supplementary material for: Altered Functional Connectivity Dynamics Serving Cognitive Flexibility in Regular Cannabis Users
Source: Addict Biol. 2025 Mar 5;30(3):e70023. doi: 10.1111/adb.70023 (PMC11881162; doi:10.1111/adb.70023)
Supplement: Supplementary file 1 — Figure S1. Average beamformer maps per condition and group. Figure S2. Whole‐brain correlation maps. [file ADB-30-e70023-s001.docx]

**Supplemental Materials**

*Altered Functional Connectivity Dynamics Serving Cognitive Flexibility in Regular Cannabis Users*

*Kellen M. McDonald, Mikki Schantell, et al.*

**Supplemental Results**


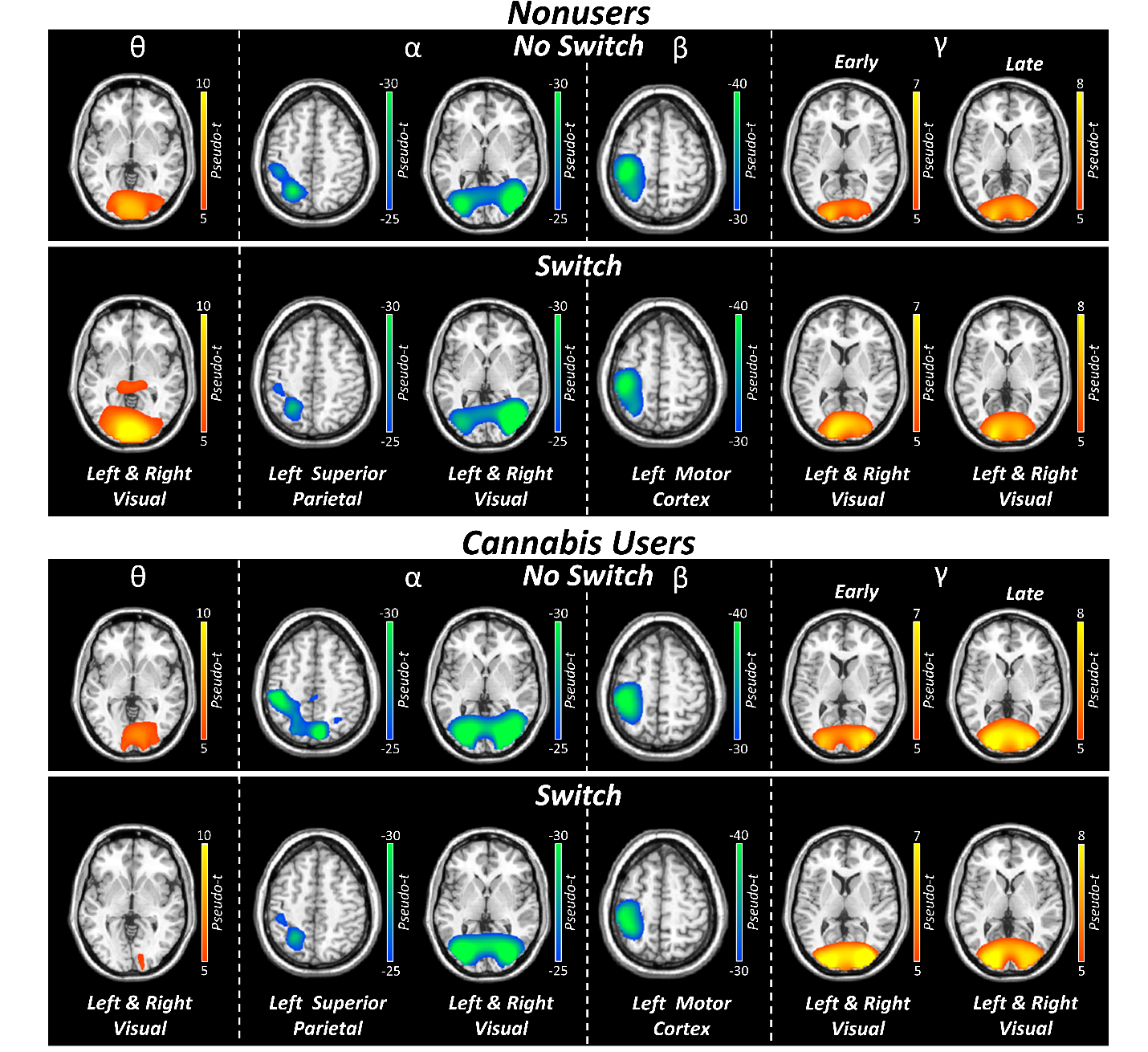


**Supplemental Figure 1. Average beamformer maps per condition and group.** Beamformer maps for each oscillatory response window are shown in pseudo-*t* units. The top two rows depict beamformer maps averaged across the nonuser group per condition, with the no-switch at the top and the switch condition directly below. The bottom two rows show maps averaged across the user group per condition, with the no-switch condition again above the switch condition. Color scale bars appear to the right of each averaged map.


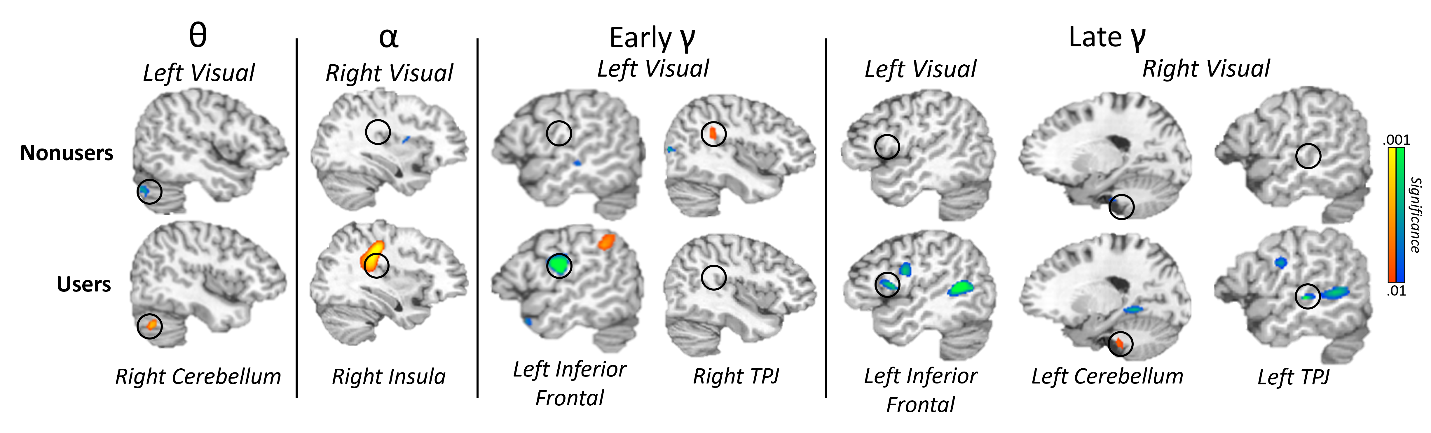


**Supplemental Figure 2. Whole-brain Correlation Maps.** The neural coherence switch cost maps were correlated with the reaction time switch cost (i.e., reaction time during the no switch condition subtracted from the switch condition). Responses are depicted separately per nonusers (top row) and users (bottom row). The circled brain regions indicate significant areas of activation after Fisher-*r*-to-*z* transformations.

**Aging Effects:**

At the request of a reviewer, we added age to each of our statistical models and recomputed them. There were no main effects or interactions with age for any of our neural measures. For behavior, only the group-by-age interaction was significant for overall task accuracy (*F* = 4.49, *p* = .041).
